# Laparoscopic vs. robotic-assisted antireflux surgery: a matched cohort analysis of procedure costs and outcomes

**DOI:** 10.1007/s00464-025-12551-1

**Published:** 2026-01-20

**Authors:** Andrés R. Latorre-Rodríguez, Arianna Vittori, Ross M. Bremner, Sumeet K. Mittal

**Affiliations:** 1 Norton Thoracic Institute, St. Joseph’s Hospital and Medical Center, 500 W Thomas Road, Phoenix, AZ 85013 USA; 2 Grupo de Investigación Clínica, Escuela de Medicina y Ciencias de La Salud—Universidad del Rosario, Bogotá, DC Colombia; 3 Department of Surgery, Oncology and Gastroenterology, School of Medicine, University of Padua, Padua, Italy; 4 School of Medicine, Creighton University, Phoenix, AZ USA

**Keywords:** Reflux, Antireflux surgery, Robotic surgery, Laparoscopic fundoplication, Robotic fundoplication, Fundoplication, Cost effectiveness, Costs

## Abstract

**Background:**

The use of robotic surgery has expanded rapidly; however, its cost-effectiveness in foregut surgery remains unclear. We aimed to compare early postoperative outcomes and procedure-related costs between robotic (R-) and laparoscopic (L-) antireflux surgery (ARS).

**Methods:**

This retrospective cohort and cost-effectiveness study was conducted using a prospectively maintained database of adults who underwent minimally invasive ARS by a single experienced esophageal surgeon. All primary elective R-ARS cases (September 2016–December 2024) were identified, and perioperative outcomes and procedure costs (US$) were compared to a 1:1 propensity-score matched L-ARS cohort. Moreover, incremental cost-effectiveness ratios (ICERs) were calculated for predefined outcomes.

**Results:**

In total, 138 patients (69 R-ARS, 69 L-ARS) were included. R-ARS was associated with a longer median operating room utilization time (169 vs 128 min., *p* < 0.001) and length-of-stay (2 vs. 1 days, *p* = 0.045) and slightly more intraoperative complications (5.8 vs. 1.4%, *p* = 0.362). Early complications (10.1% both), ICU admissions (R-ARS, 2.9% vs. L-ARS, 1.4%, *p* > 0.999), and 90-day readmissions (R-ARS, 6.1% vs. L-ARS, 3.3%, *p* = 0.749) were similar between the groups. The median all-inclusive cost was higher with R-ARS ($15,676.1 vs. $7694.9, *p* < 0.001). Although the incidence of patient-reported postoperative dysphagia was similar after R-ARS or L-ARS (26.1 vs. 30.4%, *p* = 0.705), resulting endoscopic interventions were more frequent after R-ARS (16/18 [88.9%] vs. 9/21 [42.9%], *p* = 0.008). The ICERs for intraoperative complications and 90-day readmissions were –$181,390.9 (favoring L-ARS) and $285,042.9 (favoring R-ARS), respectively.

**Conclusion:**

Overall, R-ARS may not offer superior short-term safety compared to L-ARS, utilizes greater resources, and appears to be less cost-effective.

**Supplementary Information:**

The online version contains supplementary material available at 10.1007/s00464-025-12551-1.

## Introduction

Since the early 2000s, robotic-assisted surgery has gained rapid acceptance across different surgical specialties, including thoracic and foregut [1, 2]. Compared to conventional laparoscopy, robotic platforms may offer several advantages for surgeons, including enhanced visualization, dexterity, instrument articulation, and ergonomics [3, 4]. Despite these possible advantages, the widespread adoption of robotic surgery remains limited by its financial burden. The initial investment for a single robotic platform ranges from $1 to $2.5 million (USD), and the cost of robotic instruments typically exceed that of their laparoscopic counterparts [5, 6]. These costs are particularly relevant in resource-constrained settings and prompt questioning of the cost-effectiveness of robotic surgical platforms [6].

In the field of foregut surgery, speculation on whether this technology improves surgical outcomes in technically demanding procedures such as antireflux surgery (ARS) has arisen [7–9]. The “robotic era” has increased attention and research on the growing experience at high-volume centers, yet whether these higher costs yield better patient outcomes remains uncertain [10]. Some early studies have suggested that robotic surgery may offer better perioperative outcomes compared to laparoscopic surgery for complex cases such as paraesophageal hernia repair [9, 11–14], some have shown an association between more complications and worse outcomes (i.e., increased length of stays) with robotic surgery [15], and others have reported comparable outcomes and safety profiles between both approaches, ultimately favoring the financial and clinical trade-offs of conventional laparoscopy if surgical volume remains constant [16–18].

Given the rapid adoption of robotic-assisted surgery for ARS and the limited availability of granular cost-effectiveness data from well-matched surgical cohorts, we aimed to compare perioperative outcomes and procedure-related costs between robotic and laparoscopic approaches.

## Patients and methods

### Study design and settings

This cost-effectiveness analysis was based on a single-center, retrospective, observational cohort study conducted at a tertiary regional healthcare center in the Southwestern United States. We compared perioperative outcomes and procedure-related costs between patients who underwent primary elective robotic antireflux surgery (R-ARS) and a propensity-score matched cohort of patients who underwent laparoscopic antireflux surgery (L-ARS) between September 2016 and December 2024 by a single foregut surgeon with over 20 years of experience. The Norton Thoracic Institute (NTI) Research Committee and the Institutional Review Board of St. Joseph’s Hospital and Medical Center, Phoenix, AZ approved the study under the NTI foregut umbrella protocol (PHXU-21-500-136-73-18, approval date: February 27, 2025), with patient consent waived due to the nature of the design. The study adhered to good practice guidelines and followed The Strengthening the Reporting of Observational studies in Epidemiology (STROBE) statement and checklist (Supplementary Material S1).

### Study population and study groups

All adult patients (> 18 years) who underwent primary elective R-ARS by the senior author (S.K.M.) during the study period were identified. To mitigate potential learning curve bias, only R-ARS cases performed after the senior author’s initial 100 robotic foregut procedures were included in this analysis. Further, patients who underwent (i) revisional surgery, (ii) emergent/urgent surgery, or (iii) antireflux procedures other than fundoplication (e.g., magnetic sphincter augmentation) or concomitant procedures (e.g., cholecystectomy, Collis gastroplasty) and those with a history of (iv) previous foregut procedures or (v) end-stage lung disease were excluded from analysis.

Further, a 1-to-1 propensity-score matched control group was created from all patients who underwent primary elective L-ARS by the same operating surgeon within the study period following the same exclusion criteria and considering five prespecified critical variables: (i) age, (ii) sex, (iii) BMI, (iv) use of mesh at the hiatus and (v) hernia size. The selection of these matching variables was guided by existing medical literature and clinical plausibility as relevant determinants of postoperative outcomes or cost increases. Supplementary Material S2 provides a detailed justification for each variable. Moreover, all procedures were performed using the same technique for crus closure and fundoplication following a standardized approach described elsewhere [19]. Of note, our approach consistently utilized interrupted sutures (as we do for L-ARS), which may differ from running barbed suture techniques frequently adopted in robotic-assisted procedures.

### Surgical outcomes and data sources

Demographic variables including sex, age, and body mass index (BMI) and surgical data, including approach, intraoperative procedures, operating room (OR) utilization time (per hospital records), operative time (i.e., from incision to skin closure), estimated blood loss, intraoperative complications, length of stay (LOS), admission, early postoperative complications, and Clavien–Dindo severity classification were extracted from a prospectively maintained database. Additional postoperative data (i.e., mortality, 30- and 90-day hospital readmissions [i.e., all non-planned patient hospitalizations or in-hospital observation for at least 24 h], and the need for interventions [e.g., endoscopic dilations and/or pyloric Botox injection] to manage ARS-related complications [particularly dysphagia and/or abdominal bloating] within the first postoperative year) were obtained through a structured review of electronic medical records conducted by a single investigator (A.L.).

### Costs and data sources

All costs are reported in USD ($).

#### Surgical procedure costs

The analysis used the actual costs of the surgery rather than the applied charges. These costs were obtained with the support of the hospital’s Business Management of Perioperative Services, and utilization of chargeable supplies was confirmed through a review of the operative notes. Procedure costs comprised: (i) surgical supplies, including chargeable supplies used during the procedure and reprocessing of those supplies; (ii) fixed direct costs, such as staff salaries and benefits, outsourced services (e.g., housekeeping), and other expenses directly related to patient care, including depreciation of medical equipment; and (iii) fixed indirect costs, encompassing supply costs and salaries in non–patient care departments (e.g., finance, human resources), outsourced services (e.g., hospital billing, external legal services), and other expenses (e.g., utilities, rent, building maintenance). Of note, all fixed costs were adjusted based on OR time (per-minute charges), and therefore reflect the total cost of OR time.

#### Hospital stay costs

Given that the cost of an overnight stay is included in the standard surgery charge, a LOS  ≤ 1 day did not incur additional hospitalization costs. However, the cost for each additional day of hospitalization was estimated at $4037.3. This estimate was derived from publicly available data on the U.S. national average cost ($26,646.3) and average length of stay (6.6 days) for surgical hospitalizations in 2022 with adjustments for inflation, as reported by the Healthcare Cost and Utilization Project (HCUP) Fast Stats Data Tools [20]. Furthermore, because surgical complications are associated with a 1.5-fold increase in direct hospital costs, according to Stokes et al.[21], the estimated cost of an ICU day was adjusted accordingly to $6055.90.

#### All-inclusive costs

The all-inclusive costs included procedure-related, hospitalization, and fixed platform-specific utilization costs associated with each surgical approach. The added utilization cost of conventional laparoscopy (i.e., including capital and maintenance costs per case) was adopted from Schwaitzberg et al. [22] and was estimated to be $197.3 (adjusted for inflation). All robotic procedures for this study were conducted using the DaVinci Xi Robotic Surgical System (Intuitive Surgical Inc., Sunnyvale, CA), and the utilization cost for the robotic platform (i.e., including capital and maintenance cost per case) was adopted from Khokari et al. [23] and estimated to be $3868.6 (adjusted for inflation). Of note, all inflation adjustments were performed to March 2025 using the online U.S. Bureau of Labor Statistics CPI Inflation Calculator [24].

### Sampling and sample size

Based on the findings of Bauerle et al. [16], which reported an absolute cost difference of ~ $6250 between the two surgical approaches, and assuming a reasonable degree of cost variability with an estimated standard deviation of ~ $10,000, a sample size calculation using a two-sample *t*-test (*α* = 0.05), targeting a statistical power of 0.80, indicated that a minimum of 40 patients per group (i.e., 80 patients in total) would be required to detect a meaningful difference in all-inclusive costs between groups. A maximum sample was obtained by convenience, including all consecutive individuals who met the predefined inclusion criteria.

### Statistical analysis

Categorical variables were reported as counts and proportions, whereas continuous variables were reported as medians and interquartile ranges unless otherwise specified. Propensity-score matching was performed using the “MatchIt” package, applying a classification and regression tree-based model to match patients 1:1 based on predefined critical variables. Balance between the groups was determined with an absolute standardized difference < 0.2. Normality of continuous data was assessed using the Kolmogorov–Smirnov test and Q–Q plots. Missing data were accounted for by adjusting denominators. Differences in prespecified surgical outcomes (i.e., perioperative morbidity) as well as costs (i.e., procedure costs, hospital stay costs, and all-inclusive costs) were assessed using Pearson’s Chi-squared and Student’s *t* tests or Mann–Whitney *U* tests according to the nature of data distribution. Statistical significance was set at a two-sided *p*-value of < 0.05. Incremental cost-effectiveness ratios (ICERs) were calculated from the provider perspective, and one-way sensitivity analyses were performed using both the observed minimum and maximum costs, as well as a ± 5% deterministic variation on the observed outcomes for each group. Results are presented for: (i) intraoperative complications and (ii) 90-day readmissions. All analyses were conducted using R version 4.3.1 (R Foundation for Statistical Computing, Vienna, Austria).

## Results

### Patient baseline characteristics

**Fig. 1:**
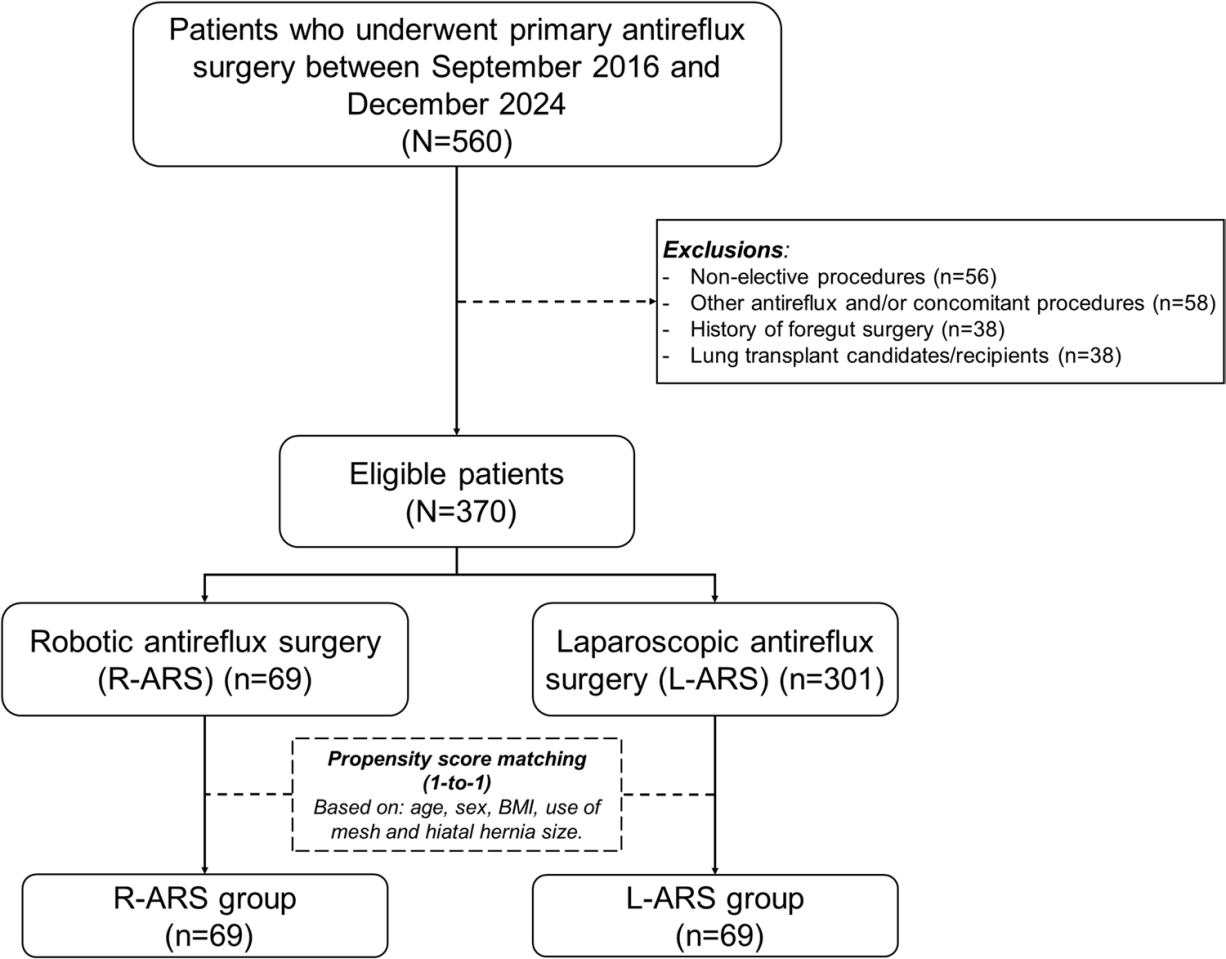
Study flow diagram

A total of 560 patients underwent primary ARS during the study period, and 190 were excluded (Fig. 1). The remaining 370 eligible patients (R-ARS, *n* = 69; L-ARS, *n* = 301) were included in the propensity-score matching process. After matching, 138 patients were included in the analysis (R-ARS, *n* = 69; L-ARS, *n* = 69). The results of the propensity-score matching and balance assessment are provided in Supplementary Material S3.

Most of the included patients were female (*n* = 101, 73.2%), the median age was 64 years [IQR 53.3–70.8], and the median BMI was 28.5 kg/m^2^ [25.4–31.5]. Except for sex distribution (females, R-ARS: 66.7% vs. L-ARS 79.7%), all critical matching variables were well balanced between groups and no significant differences were observed among demographic or surgical characteristics (Table 1).

**Table 1:** Baseline demographic and surgical characteristics

Covariate	Robotic ARS (*n* = 69)	Laparoscopic ARS (*n* = 69)	*p*-value | (SMD)*
Demographics			
Sex, female	46 (66.7)	55 (79.7)	0.124 | (0.298)
Age, years	65 [53–69.8]	63 [54–71]	0.751 | (0.033)
BMI, kg/m^2^	28.8 [25.4–31.7]	28.4 [25.3–31.4]	0.835 | (0.060)
Surgical characteristics			
Conversion to open	0 (0)	0 (0)	> 0.999
Mesh reinforcement	11 (15.9)	14 (20.3)	0.658 | (0.113)
Fundoplication type			
Nissen fundoplication	0 (0)	1 (1.4)	0.674
Toupet fundoplication	61 (88.4)	61 (88.4)	
Dor fundoplication	5 (7.2)	3 (4.3)	
Angle of His fundoplasty	3 (4.3)	3 (4.3)	
None	0 (0)	1 (1.4)	
Stomach in the thorax, %	33 [10–50]	35 [10–60]	0.915 | (0.041)
Crus antero-posterior dimension, cm	4 [4–4.5]	4 [4–4.9]	0.138
Crus transverse dimension, cm	3 [2.8–3.8]	3 [2.5–3]	0.361

Data reported as no (%) or median [IQR]

*The SMD is reported only for pre-specified critical variables used for propensity-matching

*ARS* antireflux surgery, *BMI* body mass index, *SMD* standardized mean difference

### Surgical outcomes and postoperative course

The median OR utilization time, which includes patient and equipment preparation, anesthesia induction, and transfer to the postoperative care area, was 169 min. [149–192] for R-ARS and 128 min. [118–143] for L-ARS (*p* < 0.001). Although no statistically significant differences were observed between approaches regarding blood loss or bleeding-related complications, the incidence of organ lacerations appeared slightly higher with the robotic platform (R-ARS: 4.3% vs. L-ARS: 0%, *p* = 0.243). These events included two cases of anterior fundoplication limb laceration and one case of distal esophageal laceration, all of which were immediately repaired during the index procedure without subsequent complications.

The immediate postoperative course was unremarkable for most patients. A total of 14 patients, 7 (10.1%) in each group, experienced a complication, most of which (*n* = 11, 78.6%) were mild (Clavien–Dindo grade ≤ 2). Although the median hospital stay was slightly longer in the robotic group (R-ARS: 2 [1–2] days vs. L-ARS: 1 [1–2] day, *p* = 0.045), no statistically significant differences were observed in ICU admission rates (R-ARS: 1.4% vs. L-ARS: 2.9%, *p* > 0.999) or discharge patterns, as all patients were discharged home. Similarly, the 30- and 90-day hospital readmission rates were comparable between groups. Although the incidence of postoperative dysphagia or bloating within the first year was similar between groups (R-ARS: *n* = 18 [26.1%] vs. L-ARS: *n* = 21, [30.4%], *p* = 0.705), the need for endoscopic interventions—including EGD with dilation and/or pyloric Botox injection—was significantly higher among R-ARS patients (R-ARS: 16/18 [88.9%] vs. L-ARS: 9/21 [42.9%], *p* = 0.008). A summary of the main intraoperative and postoperative outcomes is presented in Table 2.

**Table 2:** Intraoperative and postoperative surgical outcomes

Covariate	Robotic ARS (*n* = 69)	Laparoscopic ARS (*n* = 69)	*p*-value
Operative times			
OR utilization time, min	169 [149–192]	128 [118–143]	** < 0.001**
Operative time, min	120 [100–150]	80 [70–92]	** < 0.001**
Intraoperative outcomes			
Estimated blood loss, mL	25 [15–50]	25 [15–25]	0.852
Intraoperative complications	4 (5.8)	1 (1.4)	0.362
Organ laceration/perforation	3 (4.3)	0 (0)	0.243
Bleeding-related complication	1 (1.4)	1 (1.4)	> 0.999
Other type of complication	0 (0)	0 (0)	> 0.999
Perioperative outcomes			
ICU admission	1 (1.4)	2 (2.9)	> 0.999
Hospital length of stay, days	2 [1–2]	1 [1–2]	**0.045**
Early postoperative complications	7 (10.1)	7 (10.1)	> 0.999
Clavien-Dindo Grade I	3 (4.3)	5 (7.2)	0.280
Clavien-Dindo Grade II	2 (2.9)	1 (1.4)	
Clavien-Dindo Grade IIIb	2 (2.9)	0 (0)	
Clavien-Dindo Grade IVa	0 (0)	1 (1.4)	
Hospital discharge			
Discharged to home	69 (100)	69 (100)	> 0.999
Transfer to other facility	0 (0)	0 (0)	
Home health	0 (0)	0 (0)	
Hospital readmissions			
30-day readmission rate*	2/68 (2.9)	4/66 (6.1)	0.649
90-day readmissions rate*	2/61 (3.3)	4/66 (6.1)	0.749
Postoperative dysphagia and endoscopic procedures (up to 1 year after ARS)^†^			
Dysphagia (any grade)	18 (26.1)	21 (30.4)	0.705
Patients requiring EGD without dilation**	2/18 (11.1)	2/21 (9.5)	
Patients requiring EGD with dilation and/or pyloric Botox injection**	16/18 (88.9)	9/21 (42.9)	**0.008**

Data reported as no (%) or median [IQR]. Bold values indicate *p*-values < 0.05

*Adjusted to patients completing a follow-up beyond the pre-specified time point

**Adjusted to patients in which any grade of dysphagia was documented in the EMR

†At the date of data analysis, 128 (92.8%) patients had reached at least 1-year follow-up

*ARS* antireflux surgery, *BMI* body mass index, *EGD* esophagogastroduodenoscopy, *EMR* electronic medical records, *SMD* standardized mean difference

### Procedure-related costs

A comparison of costs between both surgical approaches is presented in Table 3. Overall, R-ARS was associated with higher expenses. The median surgical supplies costs (R-ARS: $2421.77 vs. L-ARS: $1495.28, *p* < 0.001), fixed direct costs (R-ARS: $762.20 vs. L-ARS: $628.50, *p* < 0.001), and fixed indirect costs (R-ARS: $6281 vs. L-ARS: $4602.90, *p* < 0.001) were all higher in the robotic group. Although the hospital costs were higher after robotic procedures, the difference was not statistically significant (*p* = 0.057); nevertheless, the median all-inclusive total cost was significantly higher for R-ARS (R-ARS: $15,676.10 vs. L-ARS: $7694.90, *p* < 0.001).

**Table 3:** Costs derived from robotic and laparoscopic antireflux surgery

Cost parameters	Robotic ARS (*n* = 69)	Laparoscopic ARS (*n* = 69)	*p*-value
Operation and hospitalization costs (US$)			
Surgical supplies	2421.77 [2391.2–4285.3]	1495.28 [770.9–2735.8]	** < 0.001**
Fixed platform utilization	3868.6	197.3	N/A
Fixed direct cost	762.2 [509.6–1222.2]	628.5 [442.4–1433.7]	** < 0.001**
Fixed indirect cost	6281.7 [4200.2–10,073.1]	4602.9 [3020.6–10,500.3]	** < 0.001**
Hospital stay	4037 [0–40,373.0]	0 [0–24,225.8]	0.057
All-inclusive total cost	15,676.1 [10,993.9–57,392.1]	7694.9 [5170.2–37,850.9]	** < 0.001**

All values are presented as median and absolute ranges [min–max]. Bold values indicate *p*-values < 0.05

*ARS* antireflux surgery, *N/A* not applicable

### Cost-effectiveness

**Fig. 2:**
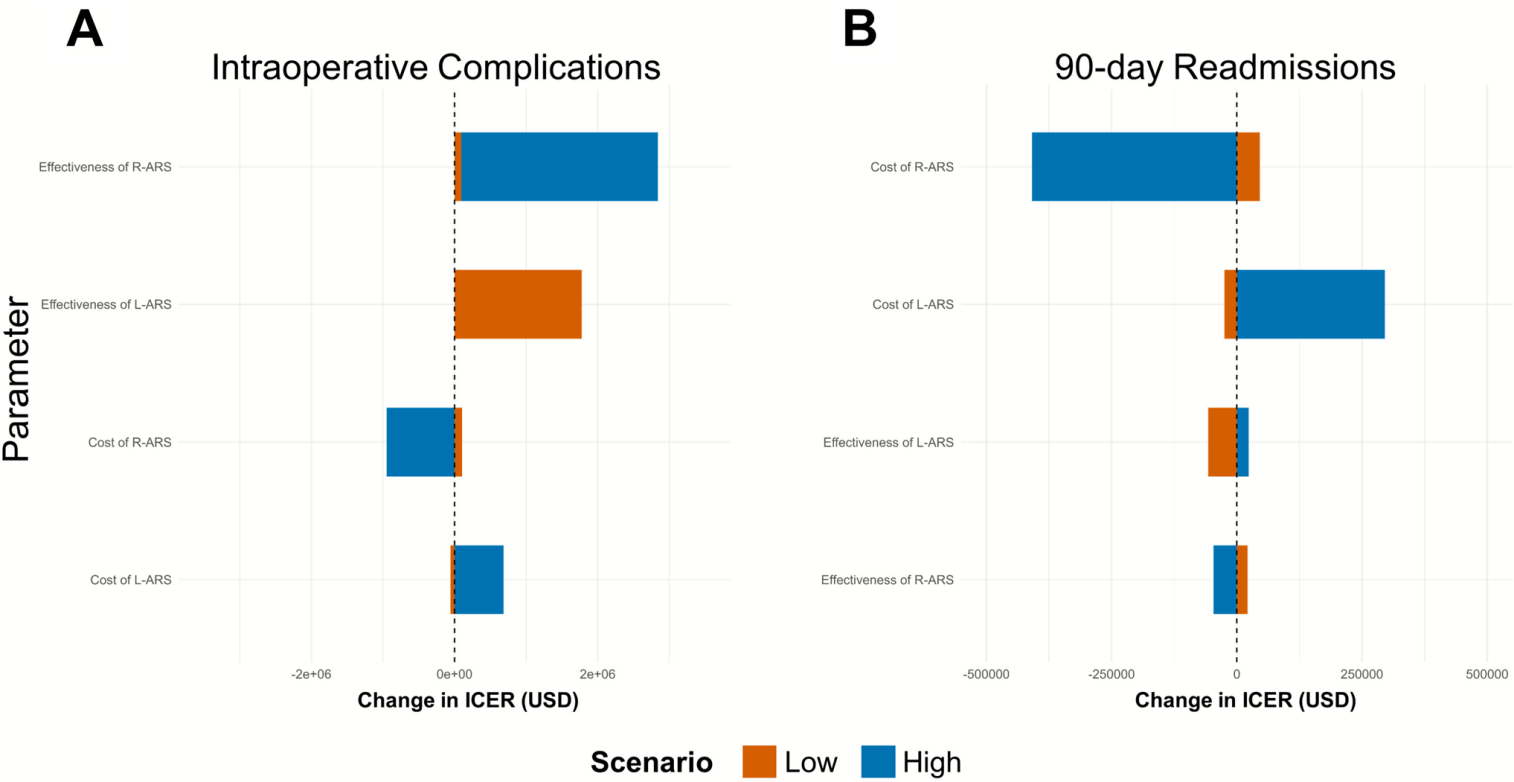
One way sensitivity analyses for outcomes of interests (tornado plots). *ICER* incremental cost-effectiveness ratio, *USD* U.S. Dollar

The ICERs and one-way sensitivity analysis results for the cost-effectiveness of the outcomes of interest are summarized in Fig. 2 and Supplementary Material S4. For intraoperative complications, the base ICER was –$181,390.9, favoring L-ARS. This negative ICER suggests that L-ARS dominates R-ARS by being both less costly and more effective at reducing intraoperative complications, even when accounting for variations in cost and effectiveness. For 90-day hospital readmissions, the base ICER was $285,042.9, indicating that although R-ARS is more expensive, it is associated with fewer readmissions. In other words, R-ARS may cost an additional $285,042.9 to prevent one hospital readmission compared to L-ARS.

## Discussion

Robotic platforms enable minimally invasive surgery for some procedures that would be otherwise challenging; [3] however, the benefits of adopting robotic-assisted surgery for benign foregut procedures (already minimally invasive with laparoscopy) remain uncertain due to the lack of high-quality, low-bias studies on patient outcomes and associated costs [2, 10]. In the present study of 138 individuals, we showed that R-ARS was associated with longer operative times, extended hospital stays, greater procedure-related costs, and increased use of hospital resources/costs while yielding similar complication and readmission rates and an even higher need for endoscopic interventions for postoperative dysphagia (increasing postoperative costs) compared to L-ARS.

Consistent with our findings, a recent meta-analysis by Gonçalves-Costa et al. [10], which included 66,725 robotic and 488,643 laparoscopic antireflux and/or hiatal hernia procedures across 14 observational studies, reported comparable perioperative outcomes between the two approaches (i.e., intraoperative complications, readmissions, and postoperative complications). Furthermore, while overall dysphagia rates during the first postoperative year were similar between groups (10.1% each)—a finding which aligns with previously reported rates [25]—patients who underwent R-ARS in our cohort had a ~ two-fold risk of requiring endoscopic therapeutic interventions. We theorize that this difference with clear clinical impact may be related to platform-specific features. Perhaps the combination of enhanced 3D-visualization and the absence of haptic feedback in the robotic platform makes it more challenging to accurately measure the tension and wrap tightness during fundoplication.

On the other hand, although a significant drawback of the meta-analysis conducted by Gonçalves-Costa et al. [10] was the wide heterogeneity between included studies (likely derived from non-granular data from national registries) and inconsistent reporting of outcomes, the authors were able to demonstrate that robotic surgery was more expensive (seven studies: 24,110 laparoscopic cases and 2394 robotic cases; standardized mean difference = 0.28, [95%CI: − 0.37, − 0.19]). Similarly, our study found that R-ARS nearly doubled the median all-inclusive cost compared to L-ARS ($15,676.1 vs. $7694.9). Moreover, since fixed costs in our study were adjusted based on OR time, we can conclude the use of the robotic platform remains more expensive even when the OR utilization and LOS were the same between the groups. These findings align with prior studies [16, 26], including one from Bauerle et al. [16], which also reported granular cost data and identified a median additional cost of approximately $6249.8 for robotic-assisted cases compared to conventional laparoscopy.

Importantly, the robotic cases included in this study were beyond the initial learning curve of the surgeon. However, to assess the potential influence of surgical experience on operative times, we performed a post-hoc analysis (not shown). For the initial ten cases, operative times were significantly longer for R-ARS than L-ARS (122.2 vs. 86.4 min, *p* < 0.01). Notably, this significant difference between platforms persisted among the last (most recent) ten cases (116.0 vs. 97.6 min, *p* < 0.05). The time for R-ARS between the first 10 and last 10 cases was 122.2 vs 116 min (*p* > 0.05). Thus suggesting that the observed differences in OR times between R-ARS and L-ARS were not predominantly driven by a learning curve effect within the study period.

From the provider perspective, our analysis (based on granular, real-world data and two clinically relevant outcomes) shows that R-ARS is generally less cost-effective than L-ARS, particularly with regard to intraoperative complications. Additionally, the benefit of R-ARS for prevention of 90-day readmissions is limited and adds substantial costs, which exceeds commonly accepted willingness-to-pay thresholds worldwide [27].

While some authors have reported a 37% overall increase in surgical volume after the adoption of robotic-assisted platforms [28], we believe that other factors including the level of care, geographic location, and insurance network affiliations play a more critical role in driving volume in foregut surgeries. Of note, the wide variability observed in the sensitivity analyses further highlights the uncertainty of R-ARS cost-effectiveness; therefore, these findings should be interpreted carefully as costs are influenced by differences in the healthcare ecosystem (hospital contracts, national and regional regulations, insurance models, etc.). Indeed, our ultimate goal with this study was to generate transparent evidence to inform clinical decision-making and support the efficient allocation of resources from the provider perspective.

Our study has certain limitations and strengths. Although the sample size was sufficient to detect differences in all-inclusive costs, it was relatively small, thus limiting more comprehensive cost-analyses and modeling. Furthermore, some of the detailed costs of the hospital stay could not be retrieved. Hence, national averages obtained from surgical hospitalizations were used, and these estimates may not accurately reflect the specific costs of care for ARS. However, surgical supply utilization was verified item-by-item, allowing for an accurate estimation of actual procedural costs rather than relying on hospital charges. Additionally, although the single-surgeon design inherently limits external generalizability, it also ensures uniformity in operative technique, intraoperative decision-making, and perioperative management, thereby improving internal validity. Of note, the five covariates included in the propensity-score matching were selected a priori based on available medical literature and clinical plausibility as determinants of both surgical approach and postoperative outcomes. Although this strategy mitigates bias in cost comparisons, residual confounding from unmeasured variables (i.e., unforeseen differences that can only be balanced with a randomized study) may have influenced postoperative outcomes. Lastly, an important strength is that all outcomes were assessed via structured chart reviews, providing a level of clinical granularity that is often missing in large-scale healthcare economic evaluations.

## Conclusion

In contrast to the evolving dogma over the last decade, the use of robotic platforms for ARS does not offer clearly superior short-term surgical outcomes compared to conventional laparoscopy. Indeed, the use of robotic-assisted surgery appears to be generally less cost-effective even in the hands of experienced surgeons. From the provider perspective, the added expense may not be justified by measurable clinical benefits. Therefore, the decision to adopt robotic surgery platforms for ARS should be guided by other factors (e.g., ergonomic benefits for surgeons, if any)—not by expectations of improved patient outcomes or cost-effectiveness. Future healthcare economic evaluations incorporating long-term outcomes and data from diverse surgical settings are highly desirable to better understand the value of robotic ARS.

## Supplementary Information

Below is the link to the electronic supplementary material.

Supplementary file1 (PDF 443 kb)
