# Supplementary material for: Laparoscopic vs. robotic-assisted antireflux surgery: a matched cohort analysis of procedure costs and outcomes
Source: Surg Endosc. 2026 Jan 20;40(4):2967–75. doi: 10.1007/s00464-025-12551-1 (PMC13053476; doi:10.1007/s00464-025-12551-1)
Supplement: Supplementary file 1 — Supplementary file1 (PDF 443 kb) [file 464_2025_12551_MOESM1_ESM.pdf]

SUPPLEMENTARY MATERIAL

Laparoscopic vs. Robotic-Assisted Antireflux Surgery: A Matched Cohort Analysis of Procedure Costs and Outcomes

Andrés R. Latorre-Rodríguez, MD[1,2]; Arianna Vittori, MD[1,3]; Ross M. Bremner, MD, PhD[1,4]; Sumeet K. Mittal, MD[1,4]

[1] Norton Thoracic Institute, St. Joseph´s Hospital, and Medical Center, Phoenix, Arizona, USA

[2] Grupo de Investigación Clínica, Escuela de Medicina y Ciencias de la Salud - Universidad del Rosario, Bogotá D.C., Colombia

[3] Department of Surgery, Oncology and Gastroenterology, University of Padua, School of Medicine, Padova, Italy

[4] School of Medicine, Creighton University, Phoenix, Arizona, USA

Table of Contents

Supplementary material S1. 2
Supplementary material S2 4
Supplementary material S3 6
Supplementary material S4 7

Supplementary material S1. STROBE Statement. Checklist of items that should be included in reports of cohort studies.

	Item No	Recommendation	Page No
**Title and abstract**	1	(*a*) Indicate the study's design with a commonly used term in the title or the abstract	Pg. 1
		(*b*) Provide in the abstract an informative and balanced summary of what was done and what was found	Pg. 2
**Introduction**			
Background/rationale	2	Explain the scientific background and rationale for the investigation being reported	Pg.4
Objectives	3	State specific objectives, including any prespecified hypotheses	Pg.4-5
**Methods**			
Study design	4	Present key elements of study design early in the paper	Pg.5-9
Setting	5	Describe the setting, locations, and relevant dates, including periods of recruitment, exposure, follow-up, and data collection	Pg.5
Participants	6	(*a*) Give the eligibility criteria, and the sources and methods of selection of participants. Describe methods of follow-up	Pg.5-6
		(*b*) For matched studies, give matching criteria and number of exposed and unexposed	Pg.8-9, Fig 1, Sup 2.
Variables	7	Clearly define all outcomes, exposures, predictors, potential confounders, and effect modifiers. Give diagnostic criteria, if applicable	Pg.6-8
Data sources/ measurement	8*	For each variable of interest, give sources of data and details of methods of assessment (measurement). Describe comparability of assessment methods if there is more than one group	Pg.6-8
Bias	9	Describe any efforts to address potential sources of bias	Pg.8-9
Study size	10	Explain how the study size was arrived at	Pg.8
Quantitative variables	11	Explain how quantitative variables were handled in the analyses. If applicable, describe which groupings were chosen and why	Pg.8-9
Statistical methods	12	(*a*) Describe all statistical methods, including those used to control for confounding	Pg.8-9
		(*b*) Describe any methods used to examine subgroups and interactions	N/A
		(*c*) Explain how missing data were addressed	Pg.9
		(*d*) If applicable, explain how loss to follow-up was addressed	Pg.9
		(*e*) Describe any sensitivity analyses	Pg.9
**Results**			
Participants	13*	(a) Report numbers of individuals at each stage of study—eg numbers potentially eligible, examined for eligibility, confirmed eligible, included in the study, completing follow-up, and analysed	Pg.9-10, Fig 1.

		(b) Give reasons for non-participation at each stage	Pg.9-10, Fig 1.
		(c) Consider use of a flow diagram	Fig 1.
Descriptive data	14*	(a) Give characteristics of study participants (eg demographic, clinical, social) and information on exposures and potential confounders	Pg.10, Tab 1
		(b) Indicate number of participants with missing data for each variable of interest	Footnote of each table
		(c) Summarise follow-up time (eg, average and total amount)	Footnote Tab 2
Outcome data	15*	Report numbers of outcome events or summary measures over time	Pg.9-12, Tab 2,3

Main results	16	(*a*) Give unadjusted estimates and, if applicable, confounder-adjusted estimates and their precision (eg, 95% confidence interval). Make clear which confounders were adjusted for and why they were included	Pg.9-12, Tab 2,3, Sup 3
		(*b*) Report category boundaries when continuous variables were categorized	Tab 2,3, Sup 3
		(*c*) If relevant, consider translating estimates of relative risk into absolute risk for a meaningful time period	N/A
Other analyses	17	Report other analyses done—eg analyses of subgroups and interactions, and sensitivity analyses	Pg.11-12, Fig 2.
**Discussion**			
Key results	18	Summarise key results with reference to study objectives	Pg.12
Limitations	19	Discuss limitations of the study, taking into account sources of potential bias or imprecision. Discuss both direction and magnitude of any potential bias	Pg.14
Interpretation	20	Give a cautious overall interpretation of results considering objectives, limitations, multiplicity of analyses, results from similar studies, and other relevant evidence	Pg.12-15
Generalisability	21	Discuss the generalisability (external validity) of the study results	Pg.14-15
**Other information**			
Funding	22	Give the source of funding and the role of the funders for the present study and, if applicable, for the original study on which the present article is based	Pg.15

*Give information separately for exposed and unexposed groups.

Note: An Explanation and Elaboration article discusses each checklist item and gives methodological background and published examples of transparent reporting. The STROBE checklist is best used in conjunction with this article (freely available on the Web sites of PLoS Medicine at http://www.plosmedicine.org/, Annals of Internal Medicine at http://www.annals.org/, and Epidemiology at http://www.epidem.com/). Information on the STROBE Initiative is available at http://www.strobe-statement.org.

Supplementary material S2. Potential confounders of surgical approach selection, operative outcomes and procedure costs.

**Variable**	**Controlled**	**Control method**	**Justification**	**References**
Age	Yes	PSM	Age could influence the development of perioperative complications, as well as be related with longer operative time, and/or higher postoperative morbidity. Moreover, age could be related with prevalence of comorbidity and ASA status.	Schietroma, M et al. (PMID: 31031314) Addo A et al. (PMID: 32170562)
Sex	Yes	PSM	To account for demographic differences and potential unknown biological responses to surgery. It has been reported that female patients are more likely to require surgical intervention.	Markar, SR et al. (PMID: 30499807)
BMI	Yes	PSM	Higher BMI is associated with technical difficulty, longer operative times, and length of stay. In rare occasions, BMI can influence the choice of robotic vs laparoscopic surgery.	Tandon A, et al. (PMID: 28853597)
Use of mesh	Yes	PSM	Mesh utilization can influence both perioperative complication risks and overall procedural cost due to additional supplies.	Stadlhuber RJ, et al. (PMID: 19067074) Latorre-Rodríguez AR, et al. (PMID: 38351425)
Hiatal hernia size	Yes	PSM	Size of the hernia is a strong independent predictor of perioperative morbidity. Moreover, in our setting it influences the surgical approach selection.	Cocco AM, et al (PMID: 36278994) Latorre-Rodríguez AR, et al. (PMID: 39722527)
Nature of procedure (elective vs. emergency)	Yes	Eligibility criteria	Urgent cases, often associated with large paraesophageal hernias and higher morbidity, typically require greater hospital resources and can influence the choice of surgical approach in our setting.	Mouroux J, et al. (PMID: 8758521) Vitelli CE, et al. (PMID: 2594262)
Type of procedure (primary vs. revisional surgery)	Yes	Eligibility criteria	Revisional cases are often more complex, which may alter surgical technique and increase the risk of postoperative complications compared with primary procedures. Moreover, in our setting it influences the surgical approach selection.	Brown AM, et al. (PMID: 31126371)
Learning curve stage	No	-	The senior author extensive laparoscopic experience (>15 years) and concurrent adoption of robotic techniques (>5 years) may have influenced early robotic outcomes, reflecting a realistic learning-phase scenario, this has been acknowledged as a study limitation.	-
Patient preference	No	-	Not systematically recorded; may have influenced approach selection in rare cases.	-

Surgeon preference	Yes	Eligibility criteria / PSM	May influence approach selection, though minimized by single-surgeon design and eligibility criteria as the surgical approach was primarily determined by: i) case urgency—laparoscopy preferred for emergencies, ii) case type—laparoscopy preferred for revisional procedures, and iii) hiatal hernia size—laparoscopy preferred for large paraesophageal or intrathoracic stomachs.	Please see the cited references provided for each covariate
Socioeconomic and/or insurance factors	No	-	Considered to present minimal influence in our setting due to uniform access to both surgical approaches.	-
Institutional scheduling / robot availability	No	-	Considered to present minimal influence in our setting due to institutional availability to both surgical approaches.	-

Abbreviations: BMI: body mass index; **PSM:** propensity score matching.

Supplementary material S3. Propensity matching results and balance assessment.

	Before propensity matching			After propensity matching	
**Covariate**	**Robotic surgery (n=69)**	**Laparoscopic surgery (n=301)**	**SMD**	**Laparoscopic surgery (n=69)**	**SMD**
Age, years	65 [53–69.8]	67 [56.9–74.7]	0.297	63 [54–71]	0.033
Sex, female	46 (66.7)	225 (74.8)	0.178	55 (79.7)	0.298
BMI, kg/m^2	28.8 [25.4–31.7]	28.3 [25.4-31.7]	0.071	28.4 [25.3–31.4]	0.060
Use of mesh	11 (15.9)	85 (28.2)	0.340	14 (20.3)	0.113
Stomach in the thorax, %	33 [10–50]	33 [10–75]	0.177	35 [10–60]	0.041

Data reported as no (%) or median [IQR].

Note: The model used the ‘*matchit’* function with a CART (Classification and Regression Trees)-based distance metric to match participants 1:1 on five pre-specified critical variables across the study groups. **Abbreviations: BMI:** body mass index; **SMD:** standardized mean difference.

Balance assessment:

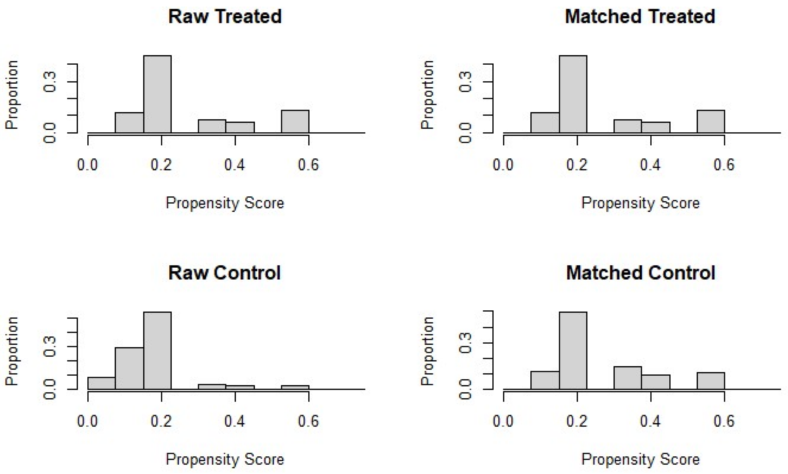

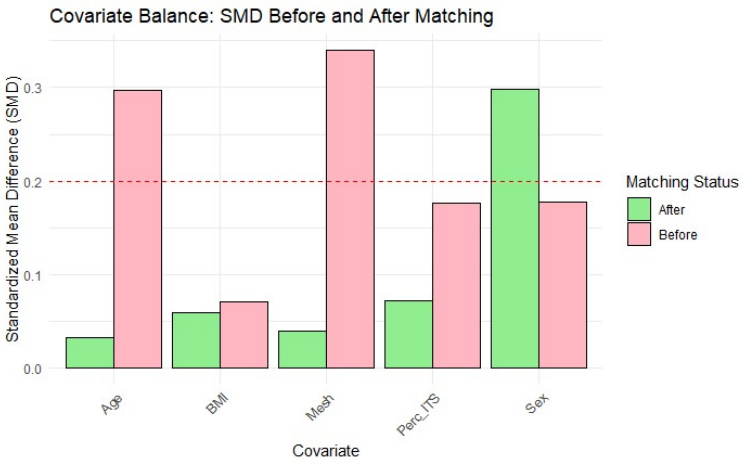

Supplementary material S4. Details of one-way sensitivity analysis for outcomes of interests.

Parameter	ICER (low)	ICER (high)	ICER (base)	Delta (low)	Delta (high)
**Intraoperative complications**					
**Cost of R-ARS**	-74,979.6	-1,129,481.8	-181,390.9	106,411.4	-948,090.91
**Cost of L-ARS**	-238,770.5	503,972.7		-57,379.6	685,363.64
**Effectiveness of R-ARS**	-867,52.2	2,660,400.0		94,638.7	2,841,790.91
**Effectiveness of L-ARS**	1,596,240.0	-137,606.9		1,777,630.9	43,784.01
**90-day hospital readmissions**					
**Cost of R-ARS**	117,825.0	1,774,900.0	285,042.9	-167,217.9	1,489,857.1
**Cost of L-ARS**	375,210.7	-791,957.1		90,167.9	-1,077,000.0
**Effectiveness of R-ARS**	-399,060.0	130,839.3		-684,102.9	-154,203.5
**Effectiveness of L-ARS**	106,416.0	-420,063.2		-178,626.9	-705,106.0

Input parameters: The minimum and maximum effectiveness values for each outcome were estimated using a ±5% range based on the observed outcomes for each group. **Abbreviations: ICER:** Incremental Cost-Effectiveness Ratio; **L-ARS:** laparoscopic antireflux surgery; **R-ARS:** robotic antireflux surgery.
